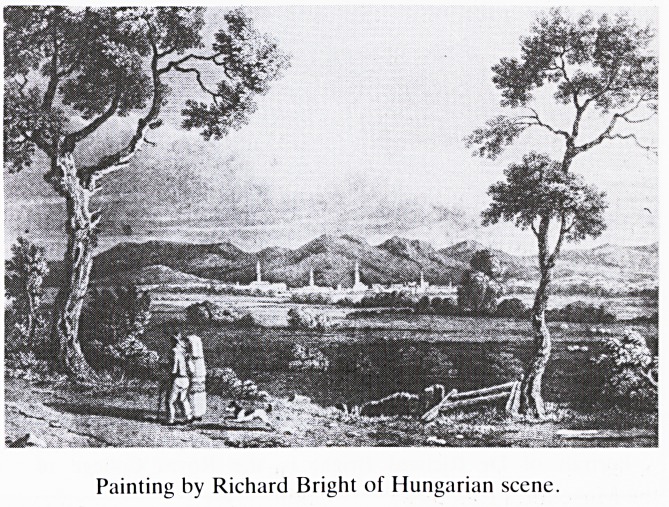# Richard Bright—A Man of Many Parts

**Published:** 1989-08

**Authors:** J. Campbell MacKenzie

**Affiliations:** Dept of Renal Medicine, Southmead Hospital


### Dr Richard Bright – A Man of Many Parts His Bicentenary Year – 1789–1858

**J. Campbell MacKenzie, R.D. B.A. F.R.C.P. (Edin)**
Dept of Renal Medicine, Southmead Hospital

Dr Richard Bright, the father of modern renal medicine, was born 200 years ago this year at 29 Queen Square, Bristol. The great medical triumvirate of Guy's Hospital in the 19th century – Hodgkin, Addison and Bright – have been immortalised by the attachment of eponyms to the diseases they decribed; however Bright although perhaps the most accomplished of the three, is less well remembered today. Many excellent accounts of his life and works have been published (1, 2, 3, 4, 5), including a biography by his great great niece, Miss Pamela Bright (6); in addition Dr Robert Kark (7) gave the FitzPatrick lecture to the Royal College of Physicians, London, in 1983 which was dedicated to Bright's memory. It is, nevertheless, appropriate, in his bicentenary year to look again at the life of this extraordinary man who always retained an association with Bristol through visits to his beloved family home at Ham Green.

His was born into a golden age of invention, of science, and of great literary, musical and artistic achievement; it was also a period of deep social unrest. The year of his birth saw the outbreak of revolution in France and the acceptance by the king of the Declaration of the Rights of Man heralding great hopes for a brighter future. Wordsworth encapsulates the feeling of the time:

Birthplace of Dr Bright—29 Queen Square.

Portrait of Dr Richard Bright in the Royal College of Physicians by F. R. Say.

> "Bliss was it in that dawn to be alive
> but to be young was very heaven . . ."

What then was the place of Bright the man, the physician, in the opening decades of a challenging 19th century?

The Brights were wealthy merchants and bankers and traditionally held high office in the city as Lord Mayor, Whig member of Parliament and Master of the Merchant Venturers. Bright's grandfather had been a founder member of the Bristol Theatre Royal and the family were keen patrons of the arts and sciences in the city.

Bright's father was a scholarly man. He was a keen amateur botanist, geologist and inventor numbering amongst close friends Joseph Priestley, James Watt, Humphry Davy and Dr. Thomas Beddoes of the Pneumatic Institute in Dowry Square, Bristol. He brought up his children in the idyllic surroundings of Ham Green and imbued his young son with a keen sense of observation coupled with an eye for detail and accurate documentation. Young Bright was at that time enrolled at the school of Dr John Estlin, a Dissenting Minister, who had some years earlier officiated at his christening at Lewins Mead Unitarian church. At the school was Henry Holland, later to become a famous physician, with whom Bright shared a lifetime's friendship.

At that time Bristol was a small, intimate community which apart from the great maritime traditions was something of a literary and artistic centre with Wordsworth, Coleridge and Southey frequent visitors and Danby, Jackson and Pocock painting scenes of the surrounding countryside and maritime activities. Not far away in the village of Berkeley, Edward Jenner was completing his work on the vaccination against smallpox.

### EDINBURGH UNIVERSITY AND TRAVELS IN ICELAND 1808–1810

After completing his education in Exeter Bright was enrolled in Edinburgh University in 1808 to undertake his premedical studies in the sciences and humanities despite his father's wish that he should enter the Ministry.

This was the Edinburgh of Walter Scott, a city of intellectuals, philosophers, and literary men; its medical school was world famous. Lodging with the Reverend Robert Morehead at 21 Hill Street, Bright quickly became acquainted with Edinburgh society. As a respite from his studies he developed his early interest in geology and met with friends at the Speculative Society which provided an excellent opportunity for the practice of oratory and debate. In 1809 he began his medical studies and wrote to his father that he had purchased a "lancet and tongue scraper" and by Christmas he was entrusted with a domiciliary midwifery case in one of Edinburgh's rat infested hovels – a chance no doubt granted by his tutor in view of the festive season!

In the Spring of 1810 his boyhood friend Henry Holland now a third year medical student arranged for them to join Sir George Steuart Mackenzie of Coul on his expedition to Iceland for the purpose of minerealogical research. By May 7th, after a rough passage from Stromness they sighted the coastline of Reykjavic "glowing like a sapphire in the distance". The time spent in Iceland provided an opportunity for Holland to study endemic diseases whilst Bright described and documented the flora and fauna of the island employing his considerable artistic skills to illustrate his work. Both young men ran a makeshift 'surgery' for the locals and Bright was pleased to find that he could at least "stitch, and treat unhealing sores."

Probably the most exciting part of the expedition for the two young men was their attempted ascent of Snaefell Jokul; this dangerous mountaineering feat nearly cost them their lives and sadly adverse weather conditions drove them back when within 100 feet of the summit – they were also cheated of the long anticipated glimpse of Greenland.

The publication of Sir George Mackenzie's book – "Travels in the Island of Iceland" (8) – brought acclaim, some particularly to Bright for his contributions. The expedition over, he returned with renewed vigour to continue his studies.

### GUY'S AND EDINBURGH 1810–1813

In October 1810 Bright began his lifelong association with Guy's Hospital which had an excellent reputation for teaching and had achieved world renown for its surgery under Astley Cooper; as such it attracted many Edinburgh medical students for their clinical years. Bright became a medical clerk to Dr Babington, whom he found to be a wise and caring physician; good manners were his hallmark in strong contrast to the often harsh and boorish behaviour of the surgeons! He had, however, an excellent teacher in Astley Cooper who was a flamboyant character, dandified in appearance but a most skilful surgeon and a droll and entertaining lecturer.

From these surgical lectures Bright developed his interest in morbid anatomy, As we have already seen he was an accomplished draughtsman and he now used his talents to record the appearances of normal and diseased organs. It was in 1811 that he first drew a granular kidney and later wrote to his father the words which in a sense expressed his medical philosophy: "For my part I am very fond of seeing." Bright was determined to raise medicine to the same status as surgery and he felt that the future of medicine depended on the ability of his generation "to do better than the last". In later years it must have given him great satisfaction to see how he and others such as Addison and Hodgkin had been successful in fulfilling that goal.

Bright's dedication to work soon won recognition and he was elected to Guy's Physical Society, to whom in 1811 he presented his first paper which was on the subject of blood letting and its limitations. That same year he addressed the Geological Society. In October 1811 he returned to Edinburgh to complete his medical studies and his doctorate on 'Contagious Erysipelas'; he received his Edinburgh M.D. in September 1813.

### DISPENSING CLINICS AND EUROPEAN TRAVEL 1814–1820

Having time to fill before starting his post at the Lock Hospital Bright entered Peterhouse, Cambridge with the intention of graduating from there also. However he found both the facilities and the course disappointing and went on to join Dr Bateman at the Caley Street Dispensary. Here he gained wide experience in general medicine and skin disorders. It was during this period he again felt the need to travel and with his father's blessing and practical help he organised a visit to Europe.

During an extended European tour in the year 1814–1815 Bright met several of the leading medical figures of the time and at the Congress of Vienna he glimpsed many of the crowned heads of Europe. For Bright, however, the most important part of this European visit was his extensive journey through Hungary which is well presented and illustrated in his book: "Travels from Vienna through Lower Hungary with some remarks on the state of Vienna during the Congress in the year 1814" (9). Bright found much to fascinate him in Hungary. The account of his visit to the baths in Buda Pesth displays a rather amusingly Victorian attitude to the mixed bathers "partially covered with linen drawers and long tresses", this in strong contrast to the 'tedium' occasioned by the visits he had made with his father to the Hotwell Baths in Bristol where feelings of pity had been the predominant emotion.

It is said that Bright did for Hungary what Byron did for Greece and this is perhaps reflected in the fact that a plaque has been unveiled at the Festetics Palace in Keszthely both to his memory and for his contribution to Hungarian culture.

On his homeward journey Bright learned of the Duke of Wellington's great victory and of course he joined the multitude visiting Waterloo. He was much moved by the suffering he witnessed and quickly became involved in caring for the wounded. He met up with old friends from Edinburgh days and with a team from Guy's sent out by Astley Cooper.

On his return from Europe Bright took up his appointment at the Lock Hospital assisting Dr John Pearson in his work on the treatment of syphilis. He was unhappy about the fact that patients with a known disease such as syphilis did not undergo post mortem: he felt that the viscera would surely be affected

Painting by Richard Bright of Hungarian scene.

and that a valuable opportunity for a deeper understanding of the disease was lost. By 1816 he had moved to the post of Assistant Physician at the Willan Fever Hospital and had obtained his Licentiate of the Royal College of Physicians.

Unfortunately Bright himself became infected with typhus and was lucky to recover, requiring an extended convalescence in Bristol and on the Continent. Later at Dr Babington's behest, he found time to address the Geological Society on his Hungarian experiences and the Medico-Chirurgical Society on European hospital practice.

### PHYSICIAN TO GUY'S HOSPITAL 1820–1844

In 1820 Bright was elected as a Fellow of the Royal Society and obtained the prestigious appointment of Assistant Physician to Guy's Hospital. It was appropriate that he should work at Guy's as it had always favoured Whigs and Dissenters. The hospital was known as the "largest repository of disease in the metropolis". He renewed his acquaintance with Dr Babington his former tutor to whose home he was often invited. Here he was to meet his future wife, Dr. Babington's daughter Martha whom he married in 1822. Tragically she died a year later five days after the birth of their son William. He overcame his grief by throwing himself with renewed vigour in to his work: it was at this time he started his magnum opus on renal and other diseases.

In 1824 Bright was appointed Full Physician to Guy's Hospital. At a later after dinner Anniversary speech Bright expressed his feelings on the appointment: "To hold the situation of Physician to Guy's Hospital is to be placed at the pinnacle of the profession"—some of us might dispute that today!

In 1827 the first volume of his "Medical Reports" was published (10). The previous year Bright had married Elizabeth Follett; they had six children. As a result of his expanding family he moved in 1831 from 14 Bloomsbury Square to 11 Saville Row where he lived for the rest of his life.

Many honours were to follow. He was elected a Fellow of the Royal College of Physicians in 1832 and became Goulstonian Lecturer at the College the following year. On the accession of Queen Victoria to the throne in 1837 he was appointed Physician Extraordinary to Her Majesty and delivered the Lumleian Lecture to the College where he was Censor from 1836 to 1839.

One of the greatest awards in 1838 was that of the Monthyon Medal of the Science Institute in Paris for his ingenious work on the kidney; it was, in its day, the equivalent of the Nobel Prize for Medicine.

### "THE MEDICAL REPORTS" AND OTHER WORK

Turning now to Bright's epoch-making discoveries and the disease that bears his name, one cannot be other than impressed that his master plan was to relate the signs and symptoms of a given disease to the underlying pathology at post mortem. Ultimately all the systems of the body would be studied and the fact that he is remembered for his seminal work on renal disease may be incidental as the vast collection of his work on other topics testifies. This systemic approach of accurately marrying clinical findings to morbid anatomy seems so simple and obvious but was not generally practised so thoroughly nor so successfully until Bright introduced it in the early 19th century. As he stated at the time "to connect accurate and faithful observations after death with symptoms displayed during life, must be in some degree to forward the object of our noble art".

"Dissection and inspection" was not always permissible and we are told that Bright often had to undertake post mortems clandestinely in order to obtain the necessary specimens; at times he was ably assisted by Hodgkin, who was Curator of the Museum. From these painstaking and extensive studies he wrote his two major works, entitled "Reports of Medical Cases selected with a view of illustrating the symptoms and cure of diseases by a reference to morbid anatomy." published by Longman and commonly known as the "Medical Reports" Volumes I & II (9, 11). The first volume appeared in 1827 and contained the now famous renal work and the second volume in 1831 dealt mainly with diseases of the brain and nervous system.

Kark in the FitzPatrick lecture has summarised Bright's work on the kidney. The discovery that by taking urine from a dropsied patient and boiling it in a teaspoon over a candle one could demonstrate albuminuria, and the correlation of this with diseased kidneys at autopsy was the basis of his life's work. However what is more significant is that he described the three main clinical presentations of acute nephritic syndrome, the nephrotic syndrome and chronic nephritis with advanced uraemia, a hard full pulse and enlarged heart with the corresponding macroscopic appearance in the kidney. From these basic findings he and his team went on to describe microscopic changes and the biochemical disturbances including a decreased excretion of urea in the urine associated with an accumulation in the blood and also the hypoalbuminaemia of the nephrotic syndrome.

As already suggested it is curious that of the three great medical men of Guy's Hospital at that time, Bright, Hodgkin and Addision, the former, although perhaps the most accomplished has become the least well-remembered and the eponym now almost never used. The reason may be that Hodgkin and Addision described single clinical entities whereas Bright's disease now covers a multitude of different syndromes and diseases.

Cameron and Becker (12) in an excellent review of the evolution of renal histology in Bright's disease drew attention to the fact that from 1839 Bright and Toynbee examined many specimens of the kidney microscopically; this work was not published possibly because of Bright's attack of acute cholecystitis in 1842.

By the 1840's the histological appearances of Bright's disease were being widely published from many sources in Britain and the Continent; one of the most important was by the famous French physician Rayer who coined the term 'Bright's Disease'.

Bright could be described as a general physician with a renal interest but there were few topics in medicine that escaped his attention. Hale-White (1921) (13) exposes the extent of Bright's knowledge of diseases other than those of the kidney and these are summarised in Table 1; in this account originality of much of Bright's work is emphasised.

**Table 1**

**Clinical syndromes and diseases other than renal described by Dr. Richard Bright.**

Localization of organic brain lesions
Dysphasia, dysgeusia and hemianopia
Jacksonian and ordinary epilepsy; spastic paraplegia
Cerebral haemorrhage, neoplasms and abscesses
Suppurative meningitis secondary to sinus and ear infections
Tuberculous meningitis and internal hydrocephalus
Subarachnoid haemorrhage from berry aneurysms

Acute yellow atrophy and cirrhosis of the liver
Jaundice in liver and gall bladder disease
Steatorrhea in pancreatic disease
Acute appendicitis, peritonitis and hydatid disease

Acute rheumatic fever, chorea, valvular disease and pericarditis
Heart block and Stoke-Adams attacks
Pertussis broncho-pneumonia*

* Bright used oxygen therapy in respiratory distress

Of Bright's many papers his most original is said to be that on abdominal diseases entitled "Cases and Observations connected with diseases of the pancreas and duodenum" (14) in which he describes fatty stools and glyclosuria in pancreatic disease. Among the many other publications it is likely that he was one of the first physicians to describe diaphragmatic hernia, acute peritonitis, acute yellow atrophy of the liver, and clubbing of the fingers in chest disease.

Bright and Addision collaborated in a textbook of medicine for students in 1839 entitled "Elements of the Practice of Medicine." (15) in which there is one of the best, if not original, descriptions of acute appendicitis. They were both excellent teachers although surprisingly we are told that Bright was not so charismatic a figure as Addison but he encouraged his students to develop delicacy in the handling of patients—"the touch of a blind man is your duty to acquire".

Before he retired from Guy's two of the most enlightened concepts introduced by Bright were the establishment of a renal unit where patients could be studied in one ward with an adjoining laboratory and the formation of a team of doctors to undertake work on renal disease. The team of assistants included Barlow, Bostock, Rees and Toynbee most of whom became well-known in their own right after his retirement. Barlow was mainly clincial while Bostock and Rees could be said to have founded clinical biochemistry following their studies on the composition of blood and urine in uraemia. Toynbee was the most interesting because not only did he collaborate with Bright between 1839 and 1842 as a gifted dissector and microscopist but later became the founder of modern otology.

During all the many years of work Bright was unstinting in his praise for his young colleagues and continued to encourage them after his retirement. Much mention is made of his unflagging energy, powers of observation, accuracy and truthfullness and his writing ability; pre-conceived ideas never tainted his descriptions.

Bright was particulalry scathing about functional diseases in his time, of which he said "this view has often been the unintentional cloak for ignorance and has materially retarded investigation". Kark refers to the lack of diaries and other sayings attributed to Bright, but he writes so clearly that in some instances one can imagine him saying it—e.g. "in disease as in other things, cause and effect will be found to follow each other in pretty regular succession".

### PRIVATE PRACTICE 1844 – 1851

Bright formally retired from Guy's in 1844 and entered full-time private practice. He had been suffering from increasing ill-health and fatigue which followed the attack of acute cholelithiasis in 1842. His recovery was slow but he was fortunate in being able to convalesce at the house of his old family friend and patient, The Hon. J. W. Croker, the Secretary of the Admiralty, whose house "Alverbank" in Alverstoke, a village near Gosport provided fine views of the Solent to the Isle of Wight: Queen Victoria often stayed there on her way across to Osborne House.

His opinion was now much sought after and his private practice increased enormously. Although he would probably want to be remembered by the many patients whom he studied in the compilation of his "Medical Reports" famous people from all walks of life consulted him. As Physician Extraordinary to Her Majesty he was entrusted in 1849 with the care of the Dowager Queen Adelaide during her last illness with lung cancer; he also attended the royal children during a measles epidemic. Lord Macaulay, Lord and Lady Jeffrey and Isambard Kingdom Brunel, who was suffering from nephritis and the poet Tennyson all sought his opinion—Tennyson was suffering from opium and alcohol addiction, he was sceptical of doctors and would not take medical advice.

One of the Bright's more interesting patients was Dr John Snow, one of the fathers of modern anaesthesia. He is also famous for having removed the handle from Broad Street Pump to quell the cholera epidemic in 1853: it is fitting that he and Bright should have founded the Epidemiological Society.

Bright had always been interested in the incidence of his disease in relation to scarlet fever and estimated that there were 500 deaths per year from nephritis in London which had a population of one million.

Bright, who had always been financially fairly independent noted at this time that his salary was around £6,000 per annum which by present day standards must be in the region of £200,000.

### THE FINAL YEARS 1851 – 1858

The last years of his life brought little respite from work and in 1851 he again felt unwell. He was suffering from nose-bleeds and diagnosed himself as having aortic valve disease. He would not allow any of his colleagues to examine him. He spent more time recovering in Argyllshire painting and fishing; he also made further visits to Europe. He made his last trip to Bristol in 1855 by train this time rather than the usual coach, to visit his brother Robert who was seriously ill. His family found time to visit the Science exhibition at the Crystal Palace but pressure of work prevented him from attending. He did however, manage to see Turner's paintings at the Royal Society of Arts.

He received many honorary memberships of medical societies throughout the world and was honoured with a Doctorate of Civil Law by Oxford University in 1853. By January 1858 he was breathless again and suffering from severe angina. He was at that time too ill to fulfil his engagements but later enjoyed a brief period of high spirits.

Bright finally died on December 16th of that year and was buried in Kensal Green. Sadly the family grave was destroyed by enemy action in 1940 during a bombing raid on London. A plaque to his memory may be seen in his parish church, St. James, Picadilly inscribed:

> "He contributed to medical science many discoveries
> and works of great value:
> and died while in full practice of his profession
> after a life of warm affection, unsullied purity
> and great usefulness".

We might also say simply that Bright was a talented, dedicated and above all an unassuming man—qualities which are perhaps most succinctly expressed in the words of the poet Wordsworth:

> "Strongest minds
> Are often those of whom the noisy world
> Hears least."

It is relatively easy to describe a man's professional achievements in his life and work but difficult to build a picture of the man himself and his character. We are told that Bright was a stocky man, short in stature with a fine head of hair and twinkling eyes. He was a good husband and father and that his many hobbies included geology, watercolour painting, travel and the study of foreign languages.

The many tributes to him after his death leave us in no doubt of his greatness. Thayer, in the Bright Oration in 1927 (16) on the occasion of the centenary of the publication of "Medical Reports" compared him with Laennec adding that although not brilliant Bright had steadfastness of purpose, an equanimity which was more precious than brilliancy; that he was straightforward, kindly, charitable, honest, tolerant and serene and through conscientious and painstaking work, became a learned and wise man earning himself a well-merited and honourable immortality

One of the greatest tributes however, came from his old friend, pupil and colleague Dr. George Hilary Barlow in 1861 (17) who said:

> "There has been no English Physician–perhaps it may be said none of any country—since the time of Harvey who has effected not only so great an advance in the knowledge of particular disease, but also some greater revolution in our habits of thought and methods of investigating morbid phenomenon and tracing aetiology of disease as the late Dr Richard Bright".

We started with a social and industrial revolution and we end with a medical one. When talking of medicine as with the flood, there is a natural watershed before Bright and after Bright.

### ACKNOWLEDGEMENTS

The author would like to thank D. Freeman Berry M.A. for assistance with the text and research and the secretaries in the Department of Renal Medicine for typing the article.

### REFERENCES

1. CHANCE, B. (1940) Richard Bright, Traveller and Artist. Bulletin of the History of Medicine. **8**, 909–933.
2. CAMERON, H. C. (1958) Richard Bright at Guy's. Guy's Hospital Reports **107**, 263–293.
3. GARRISON, F. H. (1912) Richard Bright's Travels in Lower Hungary: A Physician's Holiday. Johns Hopkins Hospital Bulletin **23**, 173–182.
4. HILL, W. (1950) Richard Bright—A Bio-Bibliography Guy's Hospital Gazette **64**, 372–379, 419–422, 439–442, 454–457 and 472–483.
5. OSMAN, A. A. (1937) Original Papers of Richard Bright on Renal Disease. Oxford University Press. London: Humphrey Milford.
6. BRIGHT, P. (1983) Dr Richard Bright (1789–1858) The Bodley Head. London.
7. KARK, R. M. (1983) The FitzPatrick Lecture: Physician Extraordinary: Richard Bright, M.D. The Royal College of Physicians of London.
8. MACKENZIE, SIR GEORGE S. (1811) Travels in the Island of Iceland. Archibald Constable: Edinburgh.
9. BRIGHT, R. (1818) Travel from Vienna through Lower Hungary; with some remarks on the state of Vienna during the Congress in the year 1814. Archibald Constable: Edinburgh.
10. BRIGHT, R. (1827) Reports of Medical Cases selected with a view of illustrating the Symptoms and Cure of Diseases with a reference to Morbid Anatomy. Vol. 1. Longman, Rees, Orme, Brown and Green. London.
11. BRIGHT, R. (1831) Reports of Medical Cases selected with a view of illustrating the Symptoms and Cure of Diseases with a reference to Morbid Anatomy. Vol. 11. Disease of the brain and nervous system. Longman, Rees, Orme, Brown and Green. London.
12. CAMERON, J. S., BECKER, E. L. (1964) Richard Bright and observations in renal histology. Guy's Hospital Reports **113**, 159–171.
13. HALE-WHITE, W. (1921) Bright's observations other than those on renal disease. Guy's Hospital Reports. **71**, 1–29.
14. BRIGHT, R. (1833) Cases and Observations connected with diseases of the pancreas and duodenum. Medico-Chirurgical Transactions, **18**, 1–56.
15. BRIGHT, R., ADDISON, T. (1839) Textbook: Elements of the Practice of Medicine. Longmans.
16. THAYER, W.S. (1927) Richard Bright: The Bright Oration delivered at Guy's Hospital of July 8th, 1927, on the occasion of the Centenary of the publication of the first vol. of Bright's Reports of Medical Cases. Guy's Hospital Reports, **77**, 253–301.
17. BARLOW, G.H. (1861) Clinical memoirs on abdominal tumours and intumescence. (Ed Barlow) New Sydenham Society.

## Figures and Tables

**Figure f1:**
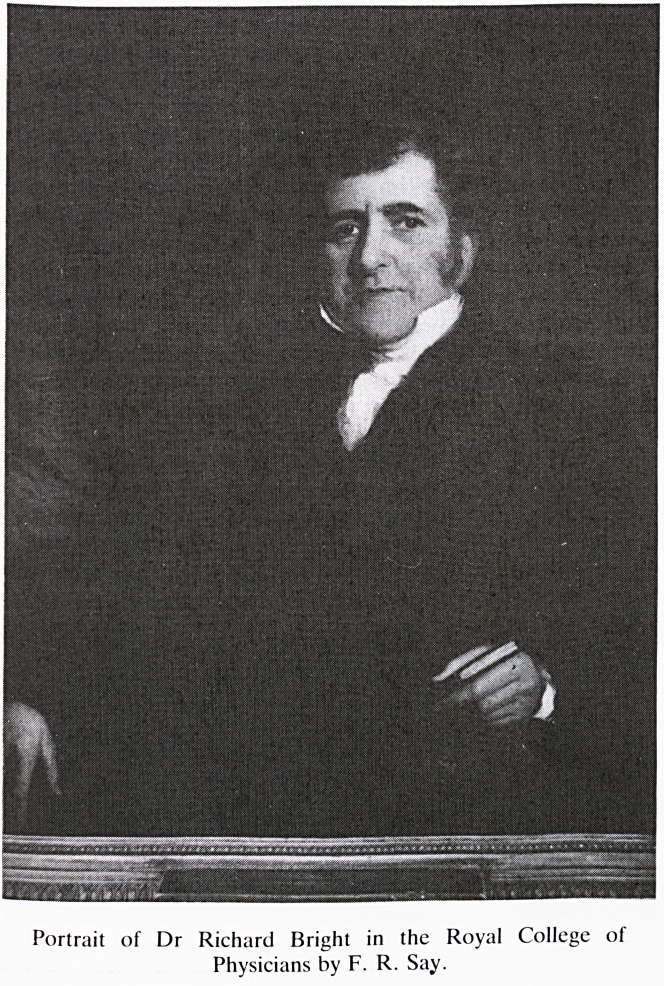


**Figure f2:**
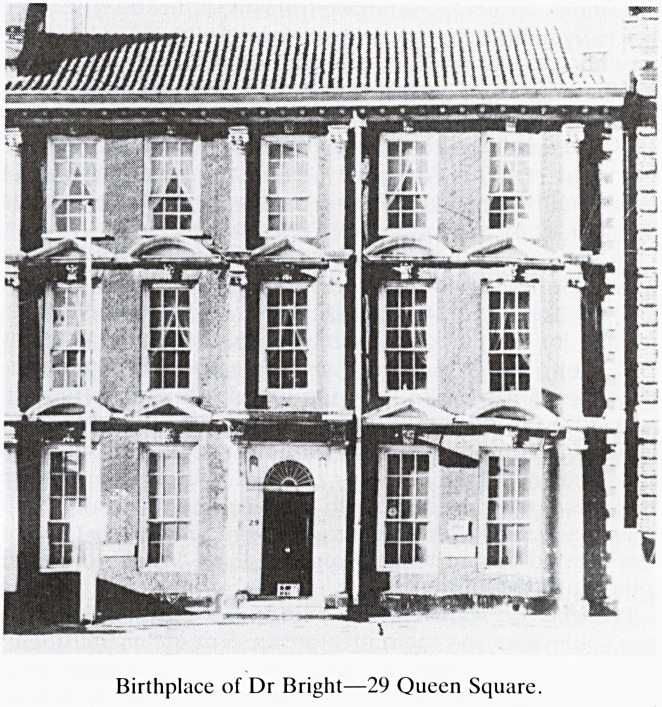


**Figure f3:**